# A Review of Cyanophage–Host Relationships: Highlighting Cyanophages as a Potential Cyanobacteria Control Strategy

**DOI:** 10.3390/toxins14060385

**Published:** 2022-05-31

**Authors:** Christopher R. Grasso, Kaytee L. Pokrzywinski, Christopher Waechter, Taylor Rycroft, Yanyan Zhang, Alyssa Aligata, Michael Kramer, Anisha Lamsal

**Affiliations:** 1 Oak Ridge Institute for Science and Education, 3909 Halls Ferry Rd, Vicksburg, MS 39180, USA; cgrasso@illumina.com; 2 Environmental Laboratory, US Army Engineer Research and Development Center, 3909 Halls Ferry Rd, Vicksburg, MS 39180, USA; taylor.e.rycroft@usace.army.mil; 3 National Oceanic and Atmospheric Administration, National Centers for Coastal Ocean Science, 101 Pivers Island Rd, Beaufort, NC 28516, USA; 4 US Bureau of Reclamation, Denver Federal Center, P.O. Box 25007, Denver, CO 80225, USA; cwaechter@usbr.gov (C.W.); aaligata@usbr.gov (A.A.); mkramer@usbr.gov (M.K.); anishalamsal@usbr.gov (A.L.); 5 Civil Engineering Department, New Mexico State University, 1780 E University Ave, Las Cruces, NM 88003, USA; zhangy@nmsu.edu

**Keywords:** cyanobacteria, cyanophage, harmful algal bloom management, phages

## Abstract

Harmful algal blooms (HABs) are naturally occurring phenomena, and cyanobacteria are the most commonly occurring HABs in freshwater systems. Cyanobacteria HABs (cyanoHABs) negatively affect ecosystems and drinking water resources through the production of potent toxins. Furthermore, the frequency, duration, and distribution of cyanoHABs are increasing, and conditions that favor cyanobacteria growth are predicted to increase in the coming years. Current methods for mitigating cyanoHABs are generally short-lived and resource-intensive, and have negative impacts on non-target species. Cyanophages (viruses that specifically target cyanobacteria) have the potential to provide a highly specific control strategy with minimal impacts on non-target species and propagation in the environment. A detailed review (primarily up to 2020) of cyanophage lifecycle, diversity, and factors influencing infectivity is provided in this paper, along with a discussion of cyanophage and host cyanobacteria relationships for seven prominent cyanoHAB-forming genera in North America, including: *Synechococcus*, *Microcystis*, *Dolichospermum*, *Aphanizomenon*, *Cylindrospermopsis*, *Planktothrix*, and *Lyngbya*. Lastly, factors affecting the potential application of cyanophages as a cyanoHAB control strategy are discussed, including efficacy considerations, optimization, and scalability for large-scale applications.

## 1. Introduction

Cyanobacteria represent the vast majority of harmful algal bloom (HAB)-causing organisms in freshwater systems. The most commonly occurring cyanobacteria HABs (cyanoHABs) include members of the genera *Microcystis*, *Dolichospermum* (formerly *Anabaena*), and *Aphanizomenon*, among others [1,2,3]. CyanoHABs are capable of negatively affecting local ecosystems and drinking water resources in a variety of ways, most notably via the production of antagonistic toxins and taste and odor compounds [4,5,6]. The frequency, duration, and geographic range of cyanoHABs are increasing in many systems due to increasing anthropogenic nutrient influxes and shifting global climates [7,8,9,10,11]. The notion that associated climate change conditions (e.g., higher temperatures, increased stratification, etc.) favor cyanobacterial dominance [7] can be tied to a range of class- to genus-specific eco-physiological traits: the unique ability of cyanobacteria to grow in warmer temperatures and regulate their buoyancy, intracellular phosphorus storage capacity, nitrogen fixation capabilities, and akinete or resting cell production, as well as their ability to adapt to variable light intensities and spectral qualities ([12] and references therein). As such, understanding the unique physiological traits of commonly occurring cyanobacteria in North America is integral to establishing effective, species-specific prevention and control measures in cyanoHAB-impaired waterways.

Both short- and long-term control solutions must be considered in HAB regulation and management. The most sustainable long-term solutions are to decrease nutrient inputs [13] and limit greenhouse gas emissions that would induce warmer climates favorable to cyanobacteria productivity [7]. However, the immediate problems cyanoHABs present necessitate short-term mitigation strategies. Current methods for mitigating cyanoHABs are generally short-lived and resource-intensive. These methods are focused on the in-water control of cyanobacteria biomass, utilizing either physical, chemical, or biological control strategies. There is a plethora of information on current scalable waterbody management resources for cyanobacteria, including a variety of physical, chemical, and biological control strategies, reviewed in depth by the US Environmental Protection Agency [14], Global Ecology and Oceanography of Harmful Algal Blooms Research Program (GlobalHAB) [15], Mitigation Subcommittee of the California Cyanobacteria and Harmful Algal Bloom Network [16], Interstate Technology and Regulatory Council [17], New England Interstate Water Pollution Control Commission [18], and Water Quality Research Australia [19]. While these methods offer short-term respite from HABs, they often introduce significant negative effects on ecosystems by impacting non-target species and may have serious consequences for ecosystem health and recovery [20,21,22,23,24]. Therefore, more targeted, species-specific approaches should be investigated with fewer negative impacts on ecosystem services.

In an effort to address these issues, the use of cyanophages (viruses that specifically target cyanobacteria) to disrupt cyanobacteria blooms prior to or during the early stages of cyanoHAB events has gained research interest. The specific targeting capabilities of cyanophages and their minimal non-target ecological effects are crucial benefits of using them to control cyanobacteria blooms. Cyanophages have varying levels of host-specificity. For example, they can infect a single strain within a species, such as Ma-LMM01 (*M. aeruginosa*—Lake Mikata *Myoviridae* 01), which infects *Microcystis Aeruginosa* strain NIES-298 [25], or they can infect multiple genera, such as one of the cyanophages found by Deng and Hayes [26] to infect members of *Dolichospermum*, *Microcystis*, and *Plantothrix*. This versatility in host-specificity is promising for the development of targeted viral control strategies that can replicate only in the presence of the target host organism. However, relevant scalable studies to validate this potential are limited.

A detailed review of research up to 2020 on cyanophages and their host relationships is provided here, with particular focus on North American freshwater cyanobacteria species and strains. Further review is provided on the natural interactions between cyanobacteria and cyanophages, highlighting natural occurrences and intra- and extracellular survivability, as well as environmental factors affecting virulence. Specific host–phage relationships for seven prominent bloom-forming genera are described. Lastly, factors affecting the potential application of cyanophages as a feasible cyanoHAB control strategy are explored, including cell and viral densities required for efficacy, and the cultivation and propagation of cyanophages for large-scale treatments.

## 2. Cyanophages

### 2.1. Life Cycle

As specialized bacteriophages, cyanophages exhibit two dominant life cycles: lytic and lysogenic. In both cases, cyanophages replicate using the host DNA machinery, which involves the following stages: attachment, penetration, biosynthesis, maturation, and release (lytic phase) [27,28]. In the lytic cycle, the mature cyanophage progeny are released after host cell lysis either through an endolysin-mediated mechanism or holin-mediated lysis ([29] and references therein). The lysogenic phage, or temperate phage, can have both lytic and/or lysogenic lifecycles. In the lysogenic cycle, cyanophage DNA is integrated into the host genome and replicated by host machinery for multiple generations to produce prophages ([30] and references therein) which are essentially in a preformed “dormant” state. Lysogenic prophages can rapidly enter the lytic cycle and be released through host cell lysis when host intracellular conditions change, such as when the host cell is stressed [28]. The lifecycle that temperate cyanophages follow depends on both intra- and extracellular factors and their interdependence, including but not limited to the impact of changing nutrient levels, ultraviolet radiation levels, and the presence of virophages (natural predators for phages), as well as any natural mutations in both the host cyanobacteria and the cyanophage [31].

### 2.2. Diversity and Specificity

Cyanophages have shown tremendous diversity in their structure, habitat and host range [32,33,34]. Numerous cyanophages have been isolated from freshwater and marine environments and are divided into three different virus families based on their morphologies: Note that viral nomenclature through the International Committee on Taxonomy of Viruses (ICTV) is moving away from morphological nomenclature, however given the breadth of studies reported herein using morphological nomenclature this was adapted throughout the review article: *Myoviridae, Podoviridae,* and *Siphoviridae* [35] (Table 1). While all cyanophages have been classified as having a single piece of double stranded DNA and the characteristic head shape of the bacteriophage, each family can be distinguished by their unique tail morphologies (Table 1). Cyanophages can also be broken down into multiple classes and sub-classes, differing in the types of cyanobacteria morphotypes and host ranges they are able to infect (Table 2) [36,37,38] (S. Where Class 1 cyanophages typically infect filamentous cyanobacteria that lack heterocysts, Class 2 cyanophages infect filamentous cyanobacteria, regardless of nitrogen fixation capabilities, and Class 3 cyanophages target unicellular or colonial cyanobacteria (Table 2). Furthermore, cyanophages can vary considerably in their host specificity, having both broad and narrow host ranges, where some cyanophages are unable to infect different strains even under the same host species, or conversely, may target multiple cyanobacteria genera [31,39,40]. For instance, the cyanophage known as Ma-LMM01 (*M. aeruginosa*—Lake Mikata *Myoviridae* 01) is only infectious to microcystin-producing *M. aeruginosa* strain NIES-298 [25]. *Podovirus* P-SSP7 is also strain-specific, infecting a single high-light-adapted *Prochlorococcus* strain out of 21 *Prochlorococcus* strains tested [41]. Additionally, Ma-LMM01, Ma-LMM02, Ma-LMM03, and Ma-HPM05 were found to specifically infect only microcystin-producing *M. aeruginosa* strains ([42] and references therein).

Similar to other viruses, cyanophages are considered an important regulator of both the abundance and composition of cyanobacteria in aquatic environments. It was found that genetic structure and diversity of cyanophages changed along water depth profiles, where maximum cyanophage diversity was correlated with maximum cyanobacterial abundances [43,44]. Furthermore, cyanophages only infect phage-sensitive cyanobacteria, which can result in the displacement of cyanophage-sensitive populations with cyanophage-insensitive populations [45]. For example, the *Microcystis*-specific phages that only infect microcystin-producing strains of *M. aeruginosa* have the potential to shift the composition of *M. aeruginosa* towards non-microcystin-producing populations, or vice versa [42].

## 3. Factors Influencing Cyanophage Infectivity

Temperature, nutrients, and irradiance are important factors affecting the stability and infectivity of cyanophages and subsequent virulence against their host. For each parameter, there are three interconnected phases that directly impact cyanophage infectivity: (1) the tolerances of the host, (2) the tolerances of the free cyanophage and (3) the propagation of the cyanophage within the host.

### 3.1. Temperature

Temperature has a profound effect on cyanobacteria propagation, which varies based on geographic location and taxa. Cyanobacteria tend to have heightened growth rates when water temperatures rise from 15 °C to 29 °C [46]. This is significant, as climate change scenarios predict that in the coming years, rivers, lakes and reservoirs will experience heightened conditions that favor cyanobacteria productivity [47]. Therefore, the ability of cyanobacteria to adapt to warming temperatures is an important consideration for future cyanophage-cyanobacteria control applications, as water temperature affects the survival rate of free cyanophages and therefore directly impacts their potential virulence.

As was observed with cyanobacteria, several studies found that cyanophage populations increased with a seasonal increase in water temperatures [48,49,50] and that their stability tended to be consistent with the stability of cyanobacteria at water temperatures up to 50 °C [37]. More specifically, at temperatures up to 40 °C, 85% of cyanophages remained virulent, while at 45 °C only 55% of cyanophages remained virulent, and at or above 50 °C, less than 0.001% of cyanophages remained virulent [36,51]. Thermotolerant cyanophage strains were able to survive at temperatures greater than 40 °C, whereas thermosensitive strains were unable to survive even at 35 °C [28]. For example, Safferman and Morris [36] and Safferman et al. [51,52] found that of the three cyanophage groups (LPP-1, SM-1, and AS-1), LPP-1 and SM-1 had the greatest temperature range, demonstrating stability between 4 °C and 40 °C. However, in LPP-1, mature particles were not formed within a host at temperatures above 31 °C. Furthermore, LPP-1 and SM-1 were inactivated at a lower temperature (55 °C) than the AS-1 group (60 °C), demonstrating various aspects of thermovariation in survivability across a diversity of cyanophages.

The infection rate of a cyanobacterium by a cyanophage is dependent upon (1) the contact rate and (2) how resistant the host cell is to the infection. Cheng et al. [53] found that cyanophages in warmer waters had more than a 50% increase in the efficiency of plaquing (EOP), which directly relates to the efficiency of cyanophage infectivity. For example, several studies have shown that an increase in water temperature led to a decrease in the water viscosity, which induced a 10.7% increase in cyanophage-host contact rate [53,54]. Furthermore, higher temperature can also lead to an increase in burst size and cyanophage adsorption on the host surface, and a decrease in the latent period [55]. Padan and Shilo [37] found that the lytic cycle could be induced under elevated temperatures, as increased temperature considerably affects cyanobacteria population homeostasis, making them more susceptible to lysis during infection. The association of the cyanophage lytic cycle with increasing temperatures is an important discovery, as global temperatures are predicted to rise owing to changing climates. Furthermore, seasonal changes could also be expected to induce cyanophages to enter the lytic cycle, which may be beneficial for operational cyanophage control scenarios. It should be noted that temperature is interrelated with pH and carbon dioxide (CO_2_), which also affect virulence; however, the connection between these parameters is still unclear [53]. There is a direct relationship between water temperature and cyanophage infectivity, but more work is needed to establish correlations, specifically with regard to host tolerances. In general, cyanophage thermotolerance studies are lacking in freshwater strains.

### 3.2. Nutrients

Macronutrients, including phosphorus, nitrogen and CO_2_ concentrations, are vital factors influencing both cyanobacteria growth and population dynamics ([56,57] and references therein). Phage proliferation strongly depends on host metabolism; host generation times affect phage latent periods and low nutrient availability results in longer latent periods and reduced burst size [58]. The metabolic status of the host is critical for viral infection and proliferation because it affects adsorption, replication, lytic activity, and survival of the phage [27]. Recently, it has been recognized that multiple nutrients may concurrently contribute to bloom occurrence [59]; however, the precise climatic and water quality conditions that trigger bloom events are still not well understood [12,60,61,62].

#### 3.2.1. Phosphorous

In the early stages of infection, cyanophages obtain the biomolecules needed to build progeny virions from the host cell and later shift to acquiring substrates that are extracellular in origin [63], which suggests that as an infection proceeds, ongoing host cell metabolism is an important factor for viral productivity [64]. It is important to note that cyanobacteria productivity is also heavily linked to extracellular nutrient concentrations, which may have even further implications for cyanophage success. For example, only 9.3% of cyanophage-infected cells lysed under limited phosphorous (P) conditions compared to 100% under replete conditions [65]. These results suggest that cyanophages became lysogenic in P-limited conditions. Continued studies have shown that, during low nutrient conditions, non-cyanophage bacteriophages enter the lysogenic phase due to unfavorable conditions for bacterial growth and production [66]. It is plausible to infer that cyanophages would function in a similar capacity given the overlap of comparable structure and function. This characteristic lifecycle shift has also been noted in cyanophages exposed to P-limited conditions, where cyanophages and their hosts can exist in an intermediate state between the lytic and lysogenic cycles, a phenomenon known as pseudolysogeny [67].

Furthermore, P-limitation in cyanobacterial host cells has also been shown to severely decrease cyanophage production rate and burst size [68,69,70]. For example, Wilson et al. [65] examined the effects of P-limitation on the cyanophage infection kinetics of S-PM2 cyanophages propagated on cultures of *Synechococcus*. Under P-limited conditions, lysis of *Synechococcus* was delayed by 18 h compared to a 9 h latent period in phosphate-replete conditions [65]. Additionally, there was an 80% reduction in burst size under P-limited conditions when *Synechococcus* was infected with cyanophage S-PM2 in comparison to replete conditions, which was also noted in a second study by Rihtman [67], who used a purified cyanophage for infection. Further, Cheng et al. [70] demonstrated significant decreases of 85% and 73% in cyanophage production rate and burst size, respectively, in P-limited *Phormidium* sp., demonstrating that P-related effects on infectivity are strongly tied to specific host–phage relationships. Cheng et al. [70] also documented increases in viral adsorption in P-limited samples from 21% to as high as 51%, further underscoring P-related effects on infectivity. Research on phage production in cyanobacteria has shown that there is a strong dependence on light and nutrient availability, but more research needs to be conducted on this topic [27].

#### 3.2.2. Nitrogen

The direct effects of nitrogen (N) on the virulence of cyanophages have not been studied extensively; however, during infection, cyanophages are known to utilize the host cell’s machinery to obtain nutrients from the extracellular medium for protein synthesis. For example, in *Synechococcus* sp. WH8102 infected with the cyanophage S-SM1, Waldbauer et al. [64] observed that proteins in progeny virion particles were composed of 41% extracellular N. Although more than half of the proteins in the phage particles were derived from the host, nutrients from the extracellular medium played an important part in viral replication. Furthermore, in a study by McKindles [71], viral replication did not occur when a strain of *Microcystis Aeruginosa* was infected with cyanophage Ma-LMM01 in N-limited media, further supporting the theory that N may be an important nutrient in phage absorption and viral replication. Lysogenic activity of cyanophages also appears to be affected by N. One study using samples of natural populations of *Synechococcus* spp. from Tampa Bay and the Gulf of Mexico showed that prophage induction is inversely correlated with the abundance of *Synechococcus*, suggesting that lysogeny may be a survival response to resource limitation [72]. This finding is further supported by another study that showed prophage production is favored over lytic behavior during periods of reduced population and vitality of *Synechococcus* spp. [66]. It should be noted that information regarding freshwater strains of *Synechococcus* spp. is lacking in this context.

#### 3.2.3. Carbon Dioxide

The effects of CO_2_ on cyanophage behavior are not as well-described in the literature as those of P or N, but it remains an important factor in phage infectivity nonetheless. Elevated dissolved CO_2_ concentrations have been shown to increase adsorption ratios as well as burst size in at least one cyanophage, coinciding with increases in host growth rate; however, there were no significant changes in the latent periods or lytic cycles between the high (740 ppm) and low (370 ppm) CO_2_ concentrations [73]. Additionally, Zhou et al. [73] documented a greater abundance of the host (*Leptolyngbya* sp.) population when cultured at the higher CO_2_ concentration compared to the lower concentration. Furthermore, it is of note that an increase in CO_2_ concentration may coincide with a decrease in environmental pH [74], and, at low pH levels, the release of cations from the culture can promote an increase in the host cell surface charge [73]; as a result, this may improve cyanophage stability and increase adsorption [75]. In a study whose findings support this, Cheng et al. [70] investigated the effects of elevated (800 µatm) CO_2_ partial pressure (pCO_2_) on cyanophages, and found a 96% increase in cyanophage production rate and a 57% increase in burst size compared to ambient (400 µatm) pCO_2_ at various host growth rates. In addition, elevated pCO_2_ resulted in a shortened latent period compared to ambient pCO_2_. In another study, during viral infection of *Synechococcus*, elevated pCO_2_ also resulted in a shortened latent period, although a decrease in burst size was observed [76]. These studies indicate that increases in CO_2_ concentration may improve infection capabilities of cyanophages by increasing their adsorption ratio and burst size. However, due to the complexity in the mechanisms involved in the host–phage relationships, additional research is necessary to investigate CO_2_ impacts on cyanophage infectivity. Furthermore, more information is needed on the combinatorial effect of changing CO_2_ concentrations alongside other factors, such as temperature, nutrients, and light conditions, particularly as the climate shifts toward warmer temperatures and as anthropogenic pollution increases.

### 3.3. Irradiance

Solar irradiance levels have been shown to directly impact cyanophages and cyanobacteria productivity, and can influence the dominant strain in cyanobacteria communities. For example, several studies have shown that toxigenic cyanobacteria species generally dominate in high-light, high-temperature, and highly stratified environments [77,78]. Alternatively, non-toxic strains thrive in a mixed water column partly because of a generally higher affinity for light absorption and unique photopigment composition [79]. Furthermore, a study by Zilliges et al. [80] highlights that this selection for toxigenic strains can be directly traced to the greater levels of ultraviolet radiation tied to a shifting climate. Collectively, this suggests that toxigenic cyanobacteria are more likely to occur in the coming years, which further drives the need to develop species/genus-specific, environmentally benign control strategies to reduce environmental and human health impacts from cyanoHABs.

Solar irradiance can also directly impact the stability of free cyanophages in aquatic systems. High solar irradiances are believed to significantly contribute to the loss of cyanophages in the natural environment as a result of impairment to phage genetic material. Specifically, the formation of pyrimidine dimers when exposed to increased irradiance has been shown to impact phage replication and infectivity [28,81], although such damage from exposure to ultraviolet light may be reversed through common photo repair mechanisms [82]. Additionally, the impact of sunlight on the rate of cyanophage decay depends on the intensity of the germicidal wavelengths that reach the cyanophage, which is impacted by the ultraviolet absorbance of the water, as well as the location of cyanophages throughout the water column [81].

Unlike other bacteriophages, light is crucial for cyanophages in the infection of cyanobacteria [83], as the adsorption and replication of some cyanophages to their host cells is light-dependent [84]. Cyanophage adsorption and replication derives most of its energy and certain resources from photosynthetic metabolism of the host cells, and is often synchronized to the light–dark cycle [83]. It was also observed that the first sign of infection is invagination of the photosynthetic lamellae, with viral particles later appearing in the space between the folded lamellae and the plasma membrane [37]. Multiple studies have also shown a heavy reliance of certain cyanophages upon the photosynthetic activity of their host cyanobacterial cells, with total losses of infectivity observed under dark conditions [85,86] and at least one cyanophage harboring a genetic homolog capable of stemming photoinhibition [87,88]. This active role of cyanophages in securing photosynthetic byproducts from their hosts further underscores the integral nature of solar irradiance to their collective success.

### 3.4. Cyanobacterial Extracellular Substances

Most cyanobacteria produce a protective boundary between themselves and the surrounding environment in the form of extracellular polymeric substances [89,90]. These substances are primarily made up of complex heteropolysaccharides, which enable cyanobacteria to dynamically regulate their extracellular glycan levels to alter mucilage complexity and function [91]. Exopolysaccharides (EPS) have many functional purposes related to their physio-chemical properties [92]. In cyanobacteria, EPS are polyanionic, forming hydrated gels that help form the scaffolding of the colony and enable metal sequestration [90]. EPS are also involved in colony formation, as they provide the “glue” that holds the individuals together into a colony.

EPS produced by cyanobacteria can act as a physical barrier to the adsorption of cyanophages, interrupting the infectivity and effectiveness of the phages [93]. EPS are known to cause lower phage mobility and even trap cyanophages [91]. Given the poor mobility of cyanophages in EPS, cyanobacteria near the outer edges of colonies and biofilms would be most susceptible to infection. As cyanobacteria colonies grow from the center outward, with the more mature cells in the center and the younger, more metabolically active cells on the edges, the majority of phage population growth in a biofilm could involve infection of bacteria that are more metabolically active, which would better support larger phage bursts [94].

Although EPS can be an effective defense strategy against bacteriophages, bacteriophages have developed mechanisms to combat them. For example, some bacteriophages can synthesize enzymes capable of degrading polymers on the cell surface of their host [95]. Some bacteriophages can also produce enzymes to depolymerize the scaffolding of the EPS and rapidly reduce the hindrance of diffusion by phages within the matrix [91]. Additionally, given the negative correlation between EPS production and cyanobacteria growth rate, EPS production is unlikely to interfere with cyanophage infectivity as it is likely to be low during active bloom events. However, more information is needed to better characterize EPS production during a HAB and its possible effects on cyanophage infectivity.

### 3.5. Summary of Environmental Factors and Their Impact on Infectivity

Several environmental factors significantly influence (1) cyanobacteria growth, (2) free cyanophage populations, and (3) cyanophage infectivity. Temperature, nutrients, and irradiance are the predominantly studied environmental parameters that have been shown to directly impact cyanophage success. Table 3 summarizes the aforementioned findings regarding the impacts of these parameters on various facets of cyanophage ecology: burst size, latent period, infectivity, adsorption, life cycle, and overall abundance. Broadly, increasing temperature coincides with an increase in all listed ecological aspects, with cyanophage life cycles being predominantly lytic in nature [48,49,50,53,55]. P-limitation resulted in decreased burst size and infectivity and an increase in latent period [65,67,68,69,70]: this limitation also drove cyanophage life cycles toward the lysogenic pathway [65,66]. It is of note that an increased P concentration has been shown to correlate with increased free cyanophage abundance, further underscoring the relationship between P and cyanophages [70]. N-limitation results were solely based on marine strains of *Synechococcus* spp. and should be explored further in freshwater systems. N-limitation drove cyanophages to lysogenic life stages and also potentially reduced adsorption and/or overall abundance [71,72]. The effects of CO_2_ on cyanophage ecology are not as well-described as the other environmental parameters discussed here; however, studies have shown that an increase in the pCO_2_ has resulted in both a decrease in latent period as well as an increase in cyanophage production [70,76]. Finally, solar irradiance is critical to the viability of host cyanobacteria cells and is therefore a major factor in cyanophage ecology. Facets such as infectivity and adsorption are strongly tied to the host cell’s photosynthetic metabolism and fluctuate alongside their host’s own optimal irradiance values [83,84]. However, it should be noted that free cyanophage abundance has an explicitly described relationship with solar irradiance, in which increased irradiance results in damage to phage genetic material [81]. The production of EPS by cyanobacteria is unlikely to provide an obstacle to the propagation of cyanophages [93], particularly as EPS production is negatively correlated with cyanobacteria growth rate [96] and growth rates are often high during bloom events, but there is limited information on EPS impacts on cyanophage infectivity, and this should be explored further. In short, understanding the effects of these critical environmental parameters on cyanophage ecology is critical to their potential operational use as a cyanoHAB control measure.

## 4. Cyanophage-Host Relationships

**Figure 1:**
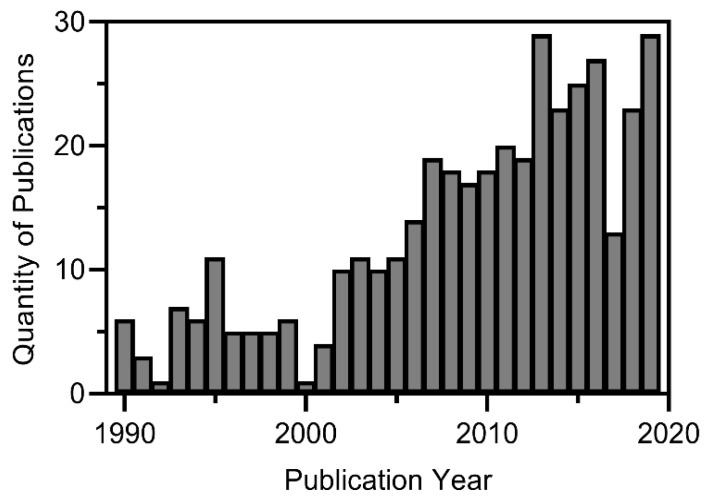
Number of publications on cyanophages from 1990 to 2019.

**Figure 2:**
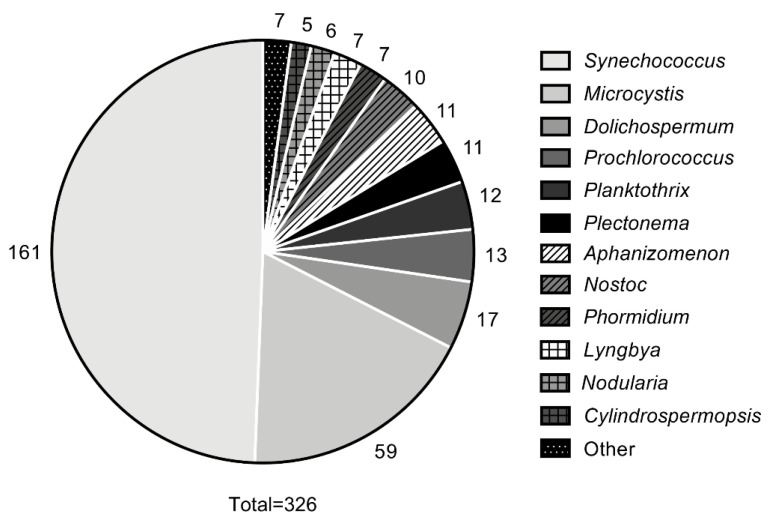
Number of publications on cyanophages by cyanobacteria host genus, from 1990 to 2019.

Before expanding upon specific documented cyanophage-host relationships, it is pertinent to discuss the history and progression of the current body of knowledge. Doing so highlights increased interest in the field, as well as demonstrates improvements that have been made in research methods key to understanding the possibilities, requirements, and barriers remaining for potential implementation. Cyanophages first appeared in the literature in 1963 [99] and have gradually increased in publication numbers over time. Between 1967 and the end of 2019 there were a total of 500 publications (either journal articles or book selections) specifically related to cyanophages, among which the majority were published after 1990 (396), with increasing numbers of publications on unique cyanophage–cyanobacteria relationships in the 2000s (Figure 1). Of the post-1990 publications, many focused on phages specific to the genera *Synechococcus* (161, 49%), *Microcystis* (59, 18%), *Dolichospermum* (17, 5%), *Prochlorococcus* (13, 4%), and *Planktothrix* (12, 4%) (Figure 2). Less prevalent in the literature were studies on the genera *Plectonema* (11, 3%), *Aphanizomenon* (11, 3%), *Nostoc* (10, 3%), *Phormidium* (7, 2%), *Lyngbya* (7, 2%), *Nodularia* (6, 2%), and *Cylindrospermopsis* (5, 2%) (Figure 2). Furthermore, few studies (7 total) were conducted on other genera (1–2 papers between 1990 and 2019) including *Anacystis* (2), *Arthrospira* (2), *Limnothrix* (1), *Synechocystis* (1), and *Trichodesmium* (1) (Figure 2). Note that some publications (18 total) covered more than one genus (up to 5 genera) and were counted for each genus they evaluated, while others (108 total) focused more broadly on cyanobacteria and were therefore excluded from the genus-level analysis.

**Figure 3:**
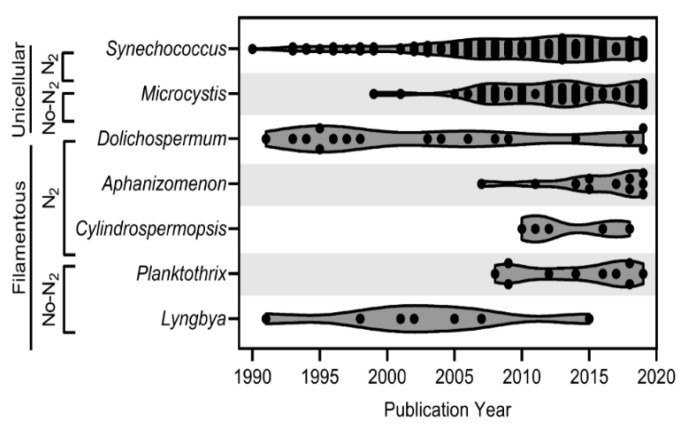
A truncated violin plot timeline for cyanophage–cyanobacteria publications by cyanobacteria genera for seven high-priority cyanobacteria genera. N_2_ signifies nitrogen fixers, and No-N_2_ denotes non-nitrogen fixers.

Throughout the period reviewed, there was a clear shift in the cyanophage-host relationships that were studied, with an overall increase in the number of publications on cyanophages starting in the early 2000s (Figure 1). Early cyanophage literature (pre-1990; 104 articles in total, including 9 papers that discussed multiple genera) was dominated by studies investigating phages specific to *Anacystis* (20, 18%), *Plectonema* (19, 17%), *Dolichospermum* (13, 12%), *Synechococcus* (12, 11%), and *Nostoc* (8, 7%) with a few articles (< 5%) on *Phormidium* (3), *Chroococcus* (1), and *Microcystis* (1) (data not shown); however, post-1990 literature was dominated by *Synechococcus*, *Microcystis,* and *Dolichospermum* (Figure 2). It is also important to note that the shift in the study of cyanophage–host relationships in recent years coincides with increases in some of the most prevalent bloom-forming cyanobacteria in North America, most notably including *Microcystis*, *Aphanizomenon*, *Cylindrospermopsis,* and *Planktothrix*. These genera featured in few publications on cyanophages pre-mid-2000s (Figure 3). The majority of publications post-1990 have been on *Synechococcus* and *Microcystis*, likely owing to their unicellular/colonial morphologies, which make them easier to work with in a laboratory setting. However, there has been a slow but steady emergence of studies on filamentous cyanobacteria of interest, including *Dolichospermum* and *Lyngbya,* dating from the early 1990s to 2019, suggesting that these organisms may serve as models for the future development of phage technologies for cyanobacteria control.

For the purposes of this review, high-priority toxic freshwater species of diverse morphologies were of interest. Commonly occurring freshwater HAB-forming cyanobacteria with toxigenic capabilities include *Microcystis, Cylindrospermopsis*, *Planktothrix* (syn. *Oscillatoria*), *Synechococcus*, *Gloeotrichia*, *Dolichospermum* (syn. *Anabaena*), *Lyngbya, Aphanizomenon*, *Nostoc*, *Schizothrix*, and *Synechocystis* [100]. Given the available literature on cyanophages and general bloom presence in North America, this review highlights cyanophage–host relationships for the following genera, all of the class Cyanophyceae: *Synechococcus*, *Microcystis*, *Dolichospermum*, *Aphanizomenon*, *Cylindrospermopsis*, *Planktothrix*, and *Lyngbya*. *Synechococcus* and *Microcystis* are unicellular and/or colonial morphotypes with variable nitrogen fixation strategies [101,102,103]. *Dolichospermum*, *Aphanizomenon,* and *Cylindrospermopsis* are filamentous cyanobacteria that have heterocysts, an indication that they are capable of fixing atmospheric N (N_2_) for energy [104,105,106]. *Planktothrix* and *Lyngbya* are also filamentous cyanobacteria, but they lack heterocysts and therefore either cannot utilize N_2_ for energy, or have developed methods evolutionarily distinct from those of heterocystous cyanobacteria [107,108,109]. Specific cyanophage–host relationships related to these high-priority cyanobacteria are described below and summarized in Table 4 to provide a greater understanding of phage technology as a potential future approach for controlling cyanobacteria in operational programs.

### 4.1. Unicellular/Colonial

#### 4.1.1. Synechococcus

*Synechococcus* (Chroococcales) is a polyphyletic alga prevalent in marine and freshwater systems [130,131]. Members are defined as picocyanobacteria, exhibiting coccoid morphology with a diameter generally < 3 µm [132], and are reportedly capable of N_2_ fixation [101,133,134]. Although the literature on picocyanobacteria toxicity is sparse, freshwater strains of *Synechococcus* have been shown to produce microcystin [135,136,137].

Several cyanophages have consistently been reported to infect freshwater *Synechococcus* strains including SM-1, SM-2, AS-1, and AS-1M [51,81,85,99,101]. Much work has been undertaken on characterizing SM-1 and SM-2 infecting the same two freshwater *Synechococcus* strains [51,81,110]. Specifically, SM-1 has been shown to be dependent upon the host cells’ photosynthetic metabolism through photosystem II (PSII) inhibition and has a latent period of 48 h [85]. Furthermore, Safferman et al. [52] later described AS-1 as a *Synechococcus*-infecting cyanophage with host specificity similar to SM-1. AS-1 was described as infecting three *Anacystis* and one *Synechococcus* strain, having a latent period of 8.5 h and average burst size of 50 plaque-forming units (PFU) per infected cell [51]. AS-1 was found to gradually inhibit electron transport within photosystem II [86] and virulence was directly linked to irradiance levels [138], similar to Mackenzie and Haselkorn [85]. A second cyanophage, AS-1M, bears a strong morphological resemblance to cyanophage AS-1, [84,139,140] but adsorbs to a host more rapidly, has a reduced latent period, and does not require NaCl as a cofactor for propagation ([139] and references therein). In addition to the well-characterized cyanophages capable of infecting *Synechococcus* belonging to SM and AS or closely related groups, a number of novel or uncharacterized cyanophages have been identified and described in the literature, and will not be discussed here [141,142,143,144,145]. Among the most well-characterized cyanophages (SM-1, SM-2, AS-1, and AS-1M), all suggest that viral infection and propagation are heavily dependent on photosynthesis, highlighting the importance of understanding how ecological factors could influence the success of future cyanophage control strategies.

#### 4.1.2. Microcystis

*Microcystis* (order Chroococcales) is perhaps the cyanobacterial genus most well-known to the general public given its ubiquity and propensity for forming toxic cyanoHABs. Members of *Microcystis* are unicellular yet colonial in nature, with varying colony size dependent upon species as well as relative dominance within a given population [146]. *Microcystis* as a genus is incapable of fixing nitrogen [147], but various species are capable of producing toxins, such as microcystin (hepatotoxin) and cyanopeptolin (neurotoxin) [148,149].

Several cyanophages in the cyanopodovirus and cyanomyovirus families have demonstrated efficacy on *Microcystis* spp. [25,96,111,112,113,114]. One of the first characterized cyanophages capable of infecting *Microcystis* spp. strains was the cyanopodovirus Ma-LBP [111]. Ma-LBP exhibited varying burst sizes between 20 and 50 PFU per cell, and achieved 95% reduction in viable host cells after 6 days. A second cyanophage Ma-LMM01 was found to be highly host-specific in its native ecology, infecting only one *M. aeruginosa* strain, exhibiting a low multiplicity of infection (MOI), and burst size up to 120 PFU per cell [25]. The genome of Ma-LMM01 had no collinearity with other cyanomyoviruses, corresponding to the high level of host specificity observed [39]. Several studies have since been published to better understand Ma-LMM01-like cyanophages on a molecular level [150,151]. Researchers have identified a highly conserved region containing a host-like *nblA* gene that plays a major role in protecting host cells during photoinhibition [42,87,88,152]. Another reported advantage for Ma-LMM01-like cyanophages is their ability to progress through various stages of infection without altering host promoter activity, which would enable the virus to shield its presence from the host [40,153]. A third cyanophage, MaMV-DC was identified as having selective infectivity towards various *Microcystis* species, with a latent period of 24–48 h and roughly 80 PFU per cell [114,154]. MaMV-DC was also identified as carrying a gene similar to host *nblA*, similarly to Ma-LMM01 [154]. Ou et al. [154] confirmed the expression of the *nblA*-like gene in the host during infection, suggesting that horizontal gene transfer or co-evolution of the *nblA* gene homolog in particular has been integral to the success of multiple cyanomyoviruses infecting *Microcystis* spp. A third cyanophage ΦMHI42 was shown to have broad spectrum activity against various cyanobacteria, including two *M. aeruginosa* strains with variable infectivity rates; *M. aeruginosa* BC84/1 stopped growing 400 h after exposure and *M. aeruginosa* CCAP 1450/8 exhibited a slowing of growth rather than outright senescence [128]. This variation in response demonstrates the importance of host specificity, particularly in optimizing the host–phage relationship to yield effective, targeted phage-mediated control.

### 4.2. Filamentous Nitrogen-Fixers

*Dolichospermum, Aphanizomenon,* and *Cylindrospermopsis* (Nostocales) are all genera representative of filamentous cyanobacteria containing N_2_-fixing heterocysts, as well as desiccation-resistant akinetes. As filamentous cyanobacteria, species are composed of multiple cells linked in strands with heterocyst and akinetes at various locations dependent on the taxa. Cell sizes vary by species, but are broadly similar across genera [155,156,157]. Anatoxin-*a*, cylindrospermopsin, and saxitoxin producers are present in all three genera [148,158], although members of *Dolichospermum* have been shown to produce a number of additional toxins and bioactive secondary metabolites, including microcystin ([149] and references therein).

#### 4.2.1. Dolichospermum

Cyanophages affecting *Dolichospermum* (formerly *Anabaena*) are some of the best-documented among filamentous cyanobacteria genera, with research extending back to the early 1970s. In the early literature (pre-1990), Currier et al. [115] evaluated the ability of the cyanomyovirus N-1 to infect two strains of *Dolichospermum*. Overall, the study found a general trend of increasing infectivity alongside increasing temperatures (as high as 51 °C) in both isolates [115]. Two additional studies in the early literature screened 2000 cyanophages and identified nine isolates (A1L-A9L) with infectivity against *D. variabilis* [116,117]. Hu et al. [117] also isolated 16 cyanophages with specificity for 11 strains of *Dolichospermum* and five strains of *Nostoc* out of a total of 69 heterocyst-forming cyanobacteria tested. In the more recent literature (post-1990), a broad study was conducted by Deng and Hayes [26] to screen 35 cyanophage isolates against various cyanobacteria hosts, including *Dolichospermum* species. A total of 16 distinct cyanophages infected at least one of the three reported *Dolichospermum* spp. strains, and seven were capable of infecting all three strains. Of these seven cyanophages with broad *Dolichospermum* sensitivity, four were cyanopodoviruses, one was a cyanomyovirus, and two had no reported taxonomy, showcasing a breadth of cyanophages with host specificity for *Dolichospermum* that could be characterized further and explored for potential biological control applications.

A study by Monegue and Phlips [118] investigated two newly isolated cyanophage strains (AC-1 and AF-1) with specificity for two strains of *Dolichospermum*. While both cyanophages were effective in reducing chlorophyll levels within their respective hosts, AF-1 showed greater efficacy during the lag or early logarithmic growth phase, and only decreased in efficacy with increasing culture age. This highlights the importance of establishing peak growth-phase efficacy when exploring cyanophages as a biological control strategy.

Several studies have explored the genomic characterization of cyanophages infecting *Dolichospermum* species [116,117,159,160,161], and have developed molecular tools to characterize cyanophage–host relationships [162,163]. Newly isolated and entirely novel cyanophage strains continue to be identified globally [26,164,165], creating a wide base from which to investigate phages effective against strains of *Dolichospermum* species, which remain a prominent HAB problem across the US.

#### 4.2.2. Aphanizomenon

The earliest descriptions of cyanophages affecting species within *Aphanizomenon* also appeared in the literature in the early 1970s [119,120]; however, clear investigations into cyanophages with laboratory propagation and evaluation became more prevalent in the last decade. To date, there have been three studies on cyanophage infectivity in the genera *Aphanizomenon* [121,122,123]; each of these explored the infectivity of Vb_AphaS-CL131 (hereafter referred to as CL131) in *A. flos-aquae*. Šulčius et al. [121] fully characterized cyanophage CL131, detailing it as a cyanosiphovirus with infectivity against *A. flos-aquae* isolated from the Curonian Lagoon (Lithuania). The infection cycle was estimated at 36 h, with cell lysis occurring after 5–7 days. CL131 was tested on a total of 18 *Aphanizomenon* strains (12 *A. flos-aquae*), five other cyanobacteria genera, and was found to infect only two strains of *A. flos-aquae* from the Curonian Lagoon, suggesting that this cyanophage is geographically bounded, as *A. flos-aquae* strains from outside of the Curonian Lagoon were not sensitive to CL131.

In a subsequent study, Šulčius et al. [122] investigated the impact of natural grazing pressures on CL131 infectivity in *A. flos-aquae* from the Curonian Lagoon. Daphnia magna was introduced to uninfected and infected *A. flos-aquae* cultures and incubated for 12 days. Šulčius et al. [122] reported that lysis-mediated and grazing-enhanced removal of shorter algal filaments resulted in a shift to longer filaments of *A. flos-aquae* more resistant to viral and grazer-related pressures. Additionally, the presence of grazers coincided with filament aggregation, which was shown to be widely insensitive to both CL131 and grazers. These are important considerations when transferring cyanophages into environmental systems.

Šulčius et al. [123] conducted a metagenomic study of CL131 with respect to similar cyanophages in other regions, including the Baltic Sea and several US waterbodies, and showed that up to 66% of CL131 proteins were conserved in Baltic Sea samples, while only 7%–20% were conserved in US samples. The studies conducted by Šulčius et al. [121,123] point to a level of specificity in CL131 that may be geographically bound, which is an important point of consideration when exploring cyanophages for control strategies that could be used to direct future research efforts in characterizing host–phage relationships and overall cyanobacteria efficacy.

#### 4.2.3. Cylindrospermopsis

Similar to the filamentous nitrogen-fixers previously described, identification of cyanophages infecting *Cylindrospermopsis* (formerly *Anabaenopsis*) occurred long before detailed studies on host–phage relationships. The earliest citation on *Cylindrospermospsis* specific cyanophages was in 1967 in India [124], identifying AR-1 as infectious to *Cylindrospermopsis raciborskii*. In 2010, Pollard and Young [125] isolated virus-like particles (VLPs) (later identified as cyanosiphoviruses) and *C. raciborskii* from a lake near Brisbane, Queensland, Australia. *C. raciborskii* was infected with the VLPs, and after 5 days biomass was reduced by 86% compared to uninfected controls. Additionally, Pollard and Young (2010) found that cell lysis generally resulted in the distribution of smaller, yet viable, fragments, suggesting that this particular virus may result in a broader distribution of *Cylindrospermopsis* if attempted as a control measure. In a second study in 2016, Steenhauer [126] characterized a novel cyanosiphovirus (CrV) from the Reeuwijkse Lakes in the Netherlands. CrV showed selective specificity for *C. raciborskii* isolated from the same lake, having full host lysis 44 h post-infection. However, CrV did not affect other cyanobacteria genera (*Aphanizomenon*, *Anabaenopsis*, *Geitlerinema*), or other *C. raciborskii* strains tested, even a second strain isolated from the same lake. Additional experiments on CrV found that both elevated irradiance and temperature resulted in a reduced latent period, and subsequently, faster achievement of host lysis, highlighting the importance of environmental factors in host–phage relationships and infectivity.

While the existing knowledge base of cyanophages affecting *Cylindrospermopsis* is limited, these studies provide some valuable insights for investigating comparable cyanophages within the US, suggesting that cyanosiphoviruses may be more selective for *Cylindospermopsis* species. Additionally, the concern proposed by Pollard and Young [125] regarding the distribution of smaller, viable filaments post-infection must be given considerable thought in transitioning cyanophages to control strategies to ensure that any future cyanoHAB control measures achieve an acceptable threshold of host mortality.

### 4.3. Filamentous Non-Nitrogen-Fixers

*Planktothrix* and *Lyngbya* (Oscillatoriales) are two bloom-forming, filamentous genera that do not possess heterocysts. Members of *Planktothrix* are capable of producing filaments a few micrometers wide and up to several millimeters long [166]. Likewise, a study by Sharp et al. [167] identified *Lyngbya* filaments as wide as 44 µm in diameter and colonies several centimeters long. Both genera have planktonic and benthic morphotypes, commonly creating mats that negatively affect benthic infauna [168]. *Planktothrix* has been shown to produce a variety of common cyanotoxins, including microcystin and anatoxin-a, while *Lyngbya* have been shown to produce lyngbyatoxin [148]. Both genera have been shown to produce saxitoxin [158].

#### 4.3.1. Planktothrix

The existing literature detailing cyanophages capable of infecting *Planktothrix* and the intricacies of their interactions was published only within the last 12 years, probably partly because of the relatively recent distinction of *Planktothrix* as its own genus [169]. Deng and Hayes [26] isolated a total of 35 cyanophages (all cyanopodoviruses or taxonomically unidentified) from Switzerland and the United Kingdom and tested them against 16 European strains of cyanobacteria, including eight strains of *Planktothrix*. Two of the eight strains were reportedly susceptible to a total of 16 distinct cyanophages, with 14 strains infecting *P. rubescens* and 13 infecting *P. agardhii*. This suggests some potential overlap in viral targets across genera, but further molecular elucidation was inconclusive.

Gao et al. [127] screened the cyanophage PaV-LD, isolated from Lake Donghu, China, against 10 cyanobacteria species, predominantly from freshwater lakes and ponds in China. Nine of the 24 strains explored were *Planktothrix*. After one week of exposure, only *P. agardhii* isolates also collected from Lake Donghu showed evidence of infection with PaV-LD, and infectivity was not observed in any other genera tested.

Watkins et al. [128] characterized infections in a number of cyanobacteria hosts using the cyanopodovirus ΦMHI42, originally isolated from *M. aeruginosa* BC84/1 (University of Bristol, UK). A total of three cyanobacteria genera (five strains) were tested, including two *Planktothrix* species. At higher MOIs, ΦMHI42 showed broad specificity, inducing signs of lytic infection in both *Microcystis* and *Planktothrix* strains after a 16-day incubation and showing greater sensitivity towards *Planktothrix,* with growth halting after five days exposure. However, similarly to the phenomenon observed by Šulčius et al. [122] with *Aphanizomenon*, clumping occurred in several infected cultures, which likely resulted in some level of resistance to infection.

In total, all reported cyanophages effective against *Planktothrix* (capable of being identified) were described as cyanopodoviruses, and exhibited a broad level of host specificity (PaV-LD notwithstanding) when compared to other cyanophages targeting filamentous cyanobacteria. If cyanophages can be adopted into control strategies, the broader host specificity may prove beneficial in targeting mixed cyanobacteria blooms in the field.

#### 4.3.2. Lyngbya

To date, there is a limited amount of literature on cyanophages infecting the genus *Lyngbya*, with only three publications since 1963 [99,118,129]. In the earliest study, Safferman and Morris [99], isolated the cyanophage LPP-1 from a waste-stabilization pond in Indiana, USA. Out of 78 organisms screened, LPP-1 lysed 11 filamentous algal strains, including two strains of *Lyngbya*. In the second study, Monegue and Phlips [118] investigated the cyanophage LW-1 and its effects on a Florida isolate of *L. wollei* over 21 days. The study demonstrated that reductions in chlorophyll concentrations were greatest (~95%) after coincubation for 14 days. In the final study by Hewson et al. [129], VLPs (similar in morphology to *Cyanosiphoviridae*) were explored against a marine strain of *L. majuscula* collected from Amity Banks, Queensland, Australia. After five days exposure, the VLPs were able to disrupt the photosynthetic machinery (via fluorescence, photochemical efficiency, and electron transport) necessary for healthy cyanobacteria growth. To assess the potential for cyanophages to control *Lyngbya,* it will be important to encourage studies on cyanophage–host specificity, given the emerging threat *Lyngbya* poses in the US and the current paucity of literature on this genus.

## 5. Cyanophages as a Control Strategy

### 5.1. Efficacy Considerations

Establishing conditions that promote efficacy is of the utmost importance when considering phages as a biological control strategy. Findings from Cheng et al. [70] affirm that phage-based control of cyanobacteria has the greatest potential for success when infective burst size, infective production rate, and adsorption are maximized, and when abortion percentage (the percentage of adsorbed cyanophages that do not lead to infection) and latent period are minimized. Another factor key to initiating cyanophage propagation for cyanoHAB management is the selection of a multiplicity of infection (MOI), which refers to the number of virions added to the number of host cells in a given treatment, such that sufficient virions are applied to exceed a threshold virion:host ratio required for self-sustained infection and propagation in the environment. However, viral loads often go unspecified in publications describing the effects of cyanophages as biological controls for cyanobacteria, obfuscating the specific cyanobacteria cell densities and associated viral densities required for effective infection and propagation. This is possibly because of the binary nature of studies searching only for positive infection or a lack thereof. Related descriptions most often cited are based on volume or the MOI. Numerous studies have used an MOI of ~1 [122,125,170] for infection. While this approximation is typical for many cyanophage infectivity experiments, there is also precedent for using significantly lower MOI values (as low as 0.1 × 10^−4^) in filamentous cyanobacteria [37,53,171]. Alternatively, it should be noted that there have been a number of studies that used a so-called “cyanophage concentrate” and added it to culture at 10% *v*/*v* [126,127,128], indicating that this imprecise practice has become something of an accepted standard in the field. Therefore, before implementing cyanophages as a biological control, it will be important to establish appropriate dosages using standardized dose-response practices to ensure reproducibility and success in the field.

It is also important to establish metrics to assess the success of cyanophages as potential biological control strategies. This is critical, as phages, in addition to having lethal impacts, can have many sub-lethal impacts that interfere with host functions in sophisticated ways and ultimately reduce cyanobacteria growth rates. Sub-lethal impacts include reduction in mechanical stiffness, change in cell shape, decrease in cell size, inhibited growth, impaired or dysregulated photosynthesis, and altered metabolism and replication [31,87,97]. For example, Jiang et al. [97] observed a reduction in mechanical stiffness in *M. aeruginosa* following infection including irregular cell shapes, cell shrinkage, and reduced membrane stiffness that contributed to inhibition of host growth and photosynthesis. Two other studies by Yoshida-Takashimia et al. [87] and Jassim and Limoges [31] identified the presence of genes related to host photosynthesis, and hypothesized that these genes might allow for phage reproduction while simultaneously stimulating photosynthesis to provide a fitness advantage for phages and maximize phage production in accordance with energy production, and limiting photoinhibition during infection [31,87]. As such, cyanophages are capable of inducing changes in the size, shape, and integrity of the host cell membrane that adversely impact nutrient uptake, while simultaneously operating host metabolic and photosynthetic pathways for further phage proliferation, actions which weaken host fitness without killing the host outright. These sub-lethal impacts may inhibit the growth of cyanobacteria and facilitate structural changes that are advantageous to competing beneficial phytoplankton in the microbial community.

When exploring cyanophages as a control strategy, there are many infection parameters that must be considered to ensure the safe and effective use of these potential biological control agents. In particular, phage propagation (reviewed in Section 3) can be a challenge in field settings given the impact of environmental conditions, such as nutrients, irradiance, and temperature on reproduction and host efficacy. The strategic application of phages alongside known environmental conditions can be used to enhance efficacy and also optimize viral propagation, though this needs to be explored further.

### 5.2. Optimization

The most critical aspect of cyanophage propagation is the health of the host cyanobacterial cells. For instance, cyanobacteria cultured in nutrient rich media have higher observed concentrations of the host global regulator RNase III and, consequently, protein nitrogen concentrations, which favor the opportunity of lytic pathway and benefit cyanophage propagation [28]. Additionally, similarly to cyanobacteria, the wide variety of cyanomyoviruses, cyanopodoviruses, and cyanosiphoviruses (formerly cyanostyloviruses) means that there are a number of parameters that must be tailored to optimize propagation of specific phages, as described in Table 3, particularly with respect to optimal host conditions that would be representative of field conditions. Laboratory cultivation of cyanobacteria has been widely documented for many decades across a multitude of studies. The most common parameters that must be taken into consideration for cyanobacterial growth are temperature, light intensity, pH, and nutrients (i.e., growth medium), although optimal ranges of each are capable of varying down to the strain level. As previously mentioned, cyanobacteria growth tends to be optimal between 15 °C and 29 °C [46]. Cultures are typically grown under a 12:12 light–dark cycle [25] and are capable of thriving under ambient outdoor light intensities (1500–2000 µE m^−2^s^−1^ on a sunny day) [172], but require far less light in laboratory culture (typically 50–200 µE m^−2^s^−1^). Both pH and nutrient concentrations are directly correlated with selected growth media; however, cyanobacteria tend to perform best under neutral to slightly alkaline conditions (pH 7–8.5) [173]. There are a wide variety of growth media available for cyanobacteria cultivation, and nutrient requirements vary by target species, but sources of nitrogen, phosphorus, iron, magnesium, trace metals, and vitamins are all required for successful cultivation [174].

### 5.3. Scalability

In utilizing cyanophages as a biological control for cyanoHABs, the most apparent issue is the matter of scale. The existing literature lacks studies describing cyanophage infectivity experiments at a mesoscale (liters), let alone the comparatively massive volumes associated with partial or full lake treatments. A study by Waechter et al. [175] conducted some preliminary hypothetical calculations on the amount of cyanophage required to treat a large freshwater cyanoHAB event. They assumed the algae was covering 80% of the lake (1500 km^2^), predominantly in the top 1 m of the water column. and assumed a MOI of 10 phages per cell, estimating that 1.2 × 10^12^ phages•L^−1^ of phage stock would be needed, equivalent to 60,000–5000 gal tanker trucks. Even when assuming only 5% of the lake was covered in cyanobacteria, a total of 395,000-gal tanker trucks of concentrated phage stock would be needed to treat the bloom area. The scalability of this technology appears to be a significant hurdle in the economic feasibility of this treatment for large events. Until a time when advancements have been made in applying phages in environmental systems, using cyanophages as a treatment for cyanoHABs may be restricted to smaller water bodies during the early stages of blooms, or as a pre-treatment to keep cyanobacteria levels low, although the continuous propagation of lytic cyanophages could potentially reduce phage volume requirements. Additional studies are needed on the environmental propagation and longevity of these viruses. There is a potential for cyanophages to be part of a suite of treatment technologies, using other control methods to make conditions in waterbodies more favorable for phage propagation and lytic activity.

## 6. Conclusions

As thoroughly reviewed here, cyanophages represent a highly specific potential method of biological control for cyanoHABs. According to the current literature, infectious cyanophages have been discovered for many of the most prominent bloom-forming cyanobacteria genera (Table 4). Although infectivity varies depending on a number of factors (Table 3), the potential remains for cyanophages to be an effective prevention and mitigation strategy in the early stages of harmful cyanoHABs. However, this review also identified several challenges that must be addressed through further laboratory studies prior to any sort of field application.

The most notable challenge relates to the issue of scalability (as covered in Section 5). The excerpt from Waechter et al. [175] clearly demonstrates that the volume of a target waterbody and the density of the cyanobacteria within it play a critical role in assessing the feasibility of applying cyanophages as a control measure. Two potential solutions to this problem may be (1) adjusting the application timetable, or (2) isolating an appropriate cyanophage with a relatively low MOI requirement (<1). By simply adjusting the application timetable to earlier in a bloom’s trajectory (e.g., pre-bloom or early stages), water resource managers would be able to suppress the growth of target cyanobacteria and reduce the concentration of required cyanophages in subsequent treatments. By identifying an appropriate cyanophage with a low MOI requirement, lower stock concentrations of phages would be needed for field applications, which is integral to the success of this technology as a standalone control measure. Although some studies described in this review employed MOI values from 1–10, there is evidence in the literature documenting infectivity with MOI values of less than 1 and as low as 1 × 10^−4^ [37,53,171]. Beyond the identification of a cyanophage with a low requisite MOI, specificity may present an additional challenge. According to the literature, host specificity can be as variable as cyanophages themselves, although many of those covered here were reported as species-specific, if not strain-specific (Table 4). Studies such as those of Ou et al. [113] and Zhong et al. [145] demonstrated a very high level of host specificity (1 of 21 and 1 of 36 strains tested, respectively); however, it should be noted that other studies, such as that of Deng and Hayes [26] reported infectivity in multiple strains of *Planktothrix* spp. Using cyanophages derived from multiple genera, including *Microcystis* and *Dolichospermum*. Cyanophages such as these, with broader ranges of host specificity, will be critical to mitigation efforts in the future. By selecting phages capable of infecting multiple genera, downstream logistics of preventative applications will become more time- and cost-effective.

Overall, the goal of this literature review was to assess the current knowledge base regarding cyanophages in order to identify factors critical to infectivity, known host–phage relationships and specificity, and challenges to the eventual utilization of cyanophages as a prevention and mitigation technique for cyanoHABs. The literature suggests that there is a diverse array of cyanophages in the environment, each exhibiting varying levels of infectivity and specificity, but effective nonetheless at neutralizing their respective target cyanobacterial strains. Additional studies are needed to identify the most appropriate cyanophages for conversion into an effective tool for water resource managers, but this review underscores that the necessary groundwork has been laid and cyanophages may play a large role in cyanoHAB management in the near future.

## Figures and Tables

**Table 1:** Cyanophage virus morphotypes by virus family summarized by Safferman et al. [35].

Virus Family	Morphology	Examples
*Myoviridae*	An isometric head separated by a neck from a long complex tail with a contractile sheath and central tube	Cyanomyovirus
*Podoviridae*	An isometric head with a short tail (without a neck), generally less than half the diameter of the widest head dimension	Cyanopodovirus
*Siphoviridae*	An isometric head with a noncontractile tail as long or longer than the diameter of the widest head dimension	Cyanosiphovirus (formerly Cyanostylovirus)

**Table 2:** Cyanophage groups categorized by known target cyanobacteria.

Cyanophage class	Groups	Known Target Cyanobacteria	Unique Cyanobacteria Traits
Class 1	LPP	*Lyngbya* *Phormidium* *Plectonema*	Filamentous, non-heterocystous
Class 2	A	*Dolichospermum*	Filamentous, both heterocystous and non-heterocystous
	N	*Dolichospermum*	
	AN	*Dolichospermum* *Nostoc*	
	NP	*Nostoc* *Plectonema*	
Class 3	AS	*Anacystis* *Synechococcus* *Microcystis*	Unicellular, colonial
	SM	*Anacystis* *Synechococcus* *Microcystis*	

**Table 3:** Summary of environmental factors and their influence on cyanophages.

	Temperature	Nutrients	EPS	Irradiance	References
Burst size	Increased with temperature.	Decreased under P-limitation. Inconsistent findings with elevated pCO_2_.			[55,65,67,70]
Latent period	Decreased with temperature.	Increased under P-limitation. Decreased under elevated pCO_2_			[55,65,76]
Infectivity	Increased in warmer waters (up to 40 °C to 45 °C).	Decreased under P-limitation.	Decreased with greater EPS production.	Decreased with high light owing to dimer formation. Light-dependent for some cyanophages.	[53,81,83,84,93]
Adsorption	Increased with temperature (e.g., shift from 24 °C to 35 °C).	Increased with elevated pCO_2_. Decreased under N-limitation.	Decreased with physical impedance of cyanophage diffusion.	Light-dependent as cyanophage adsorption derives much if its energy from host photosynthesis.	[55,71,84,91]
Life cycle	Driven toward lytic with increasing temperature.	Driven toward lysogenic under P- and N-limitation.		Driven toward lytic with increasing irradiance for some cyanophages.	[55,65,66,72,97]
Abundance	Increased with temperature.	Increased free cyanophage in heightened P conditions. Increased production with elevated pCO_2_. No change in replication within host. Decreased under N-limitation.		Decreased due to inactivation from extended exposure to germicidal UV wavelengths.	[48,49,50,69,70,71,81,98]

N-limitation information is for marine strains of Synechococcus as this information is lacking for freshwater strains. Note that marine and freshwater strains are not distinguished here but this may play a role in further elucidating environmental factors influencing infectivity.

**Table 4:** Summary of identified host–phage relationships.

Cyanobacteria Genus	Identified Cyanophages	Range of Specificity	Candidate for Broad HAB Control	References
*Synechococcus*	SM-1, SM-2, NRC-1, AS-1, AS-1M, S-CRM01, S-EIV1, S-LBS1	Strain-to-Species-Level	Poor; primarily strain-specific	[51,81,85,99,110]
*Microcystis*	Ma-LBP, Ma-LMM01, MaMV-DC, ΦMHI42	Strain-to-Genus-Level	Fair; some phages infectious across multiple species	[25,96,111,112,113,114]
*Dolichospermum*	N-1, A-1L, A-4L, AC-1, AF-1, AN-10, AN-13, AN-23, M-CP1/2/3, A-CP1/2/3/4, A-CP6/7, A-CM1/2, A-CF1, A-CS1/2	Strain-to-Class-Level	Good; multiple phages infectious in multiple genera	[26,115,116,117,118]
*Aphanizomenon*	Vb_AphaS-CL131	Strain-Level	Poor; strain-specific	[119,120,121,122,123]
*Cylindrospermopsis*	AR-1, CrV	Strain-Level	Poor; strain-specific	[124,125,126]
*Planktothrix*	PaV-LD, M-CP5/6, A-CP1/4, P-Z1 through P-Z12	Strain-to-Class-Level	Fair; some phages infectious in *Dolichospermum* spp.	[26,127,128]
*Lyngbya*	LPP-1, LW-1	Strain-to-Class-Level	Good; LPP-1 infectious in multiple filamentous genera	[99,118,129]

## Data Availability

The data presented in this study are not publicly available but are available on request from the corresponding author.

## References

[1] Carmichael W.W. (2001). Health Effects of Toxin-Producing Cyanobacteria: “The CyanoHABs”. Hum. Ecol. Risk Assess. Int. J..

[2] Paerl W.H., Otten T.G. (2013). Harmful cyanobacterial blooms: Causes, consequences, and controls. Environ. Microbiol..

[3] Ko S.-R., Srivastava A., Lee N., Jin L., Oh H.-M., Ahn C.-Y. (2019). Bioremediation of eutrophic water and control of cyanobacterial bloom by attached periphyton. Int. J. Environ. Sci. Technol..

[4] Funari E., Testai E. (2008). Human Health Risk Assessment Related to Cyanotoxins Exposure. Crit. Rev. Toxicol..

[5] Metcalf J.S., Codd G.A., Whitton B. (2012). Cyanotoxins. Ecology of Cyanobacteria II.

[6] Suurnäkki S., Gomez-Saez G.V., Ylinen A.H., Jokela J., Fewer D., Sivonen K. (2015). Identification of geosmin and 2-methylisoborneol in cyanobacteria and molecular detection methods for the producers of these compounds. Water Res..

[7] Mooij W.M., Hülsmann S., Domis L.N.D.S., Nolet B.A., Bodelier P.L., Boers P.C., Pires L.M.D., Gons H.J., Ibelings B.W., Noordhuis R. (2005). The impact of climate change on lakes in the Netherlands: A review. Aquat. Ecol..

[8] Codd G.A., Morrison L.F., Metcalf J. (2005). Cyanobacterial toxins: Risk management for health protection. Toxicol. Appl. Pharmacol..

[9] Bláha L., Babica P., Maršálek B. (2009). Toxins produced in cyanobacterial water blooms—Toxicity and risks. Interdiscip. Toxicol..

[10] O’Neil J.M., Davis T.W., Burford M.A., Gobler C.J. (2012). The rise of harmful cyanobacteria blooms: The potential roles of eutrophication and climate change. Harmful Algae.

[11] Huisman J., Codd G.A., Paerl H.W., Ibelings B.W., Verspagen J.M.H., Visser P.M. (2018). Cyanobacterial blooms. Nat. Rev. Genet..

[12] Carey C.C., Ibelings B.W., Hoffmann E.P., Hamilton D.P., Brookes J.D. (2012). Eco-physiological adaptations that favour freshwater cyanobacteria in a changing climate. Water Res..

[13] Boesch F.D., Anderson D.M., Horner R.A., Shumway S.E., Tester P.A., Whitledge T.E. (1997). Harmful Algal Blooms in Coastal Waters: Options for Prevention, Control, and Mitigation.

[14] USEPA (United States Environmental Protection Agency) (2020). Control Measures for Cyanobacterial HABs in Surface Water. https://www.epa.gov/cyanohabs/control-measures-cyanobacterial-habs-surface-water.

[15] Burford A.M., Gobler C.J., Hamilton D.P., Visser P.M., Lurling M., Codd G.A. (2019). Solutions for Managing Cyanobacterial Blooms: A Scientific Summary for Policy Makers.

[16] California Water Quality Monitoring Council (2020). Algae Mitigation Technique Selection Process for Lakes. https://mywaterquality.ca.gov/habs/resources/docs/flow_chart_draft_20190515.pdf.

[17] ITRC (Interstate Technology and Regulatory Council) (2020). Strategies for Preventing and Managing Harmful Cyanobacterial Blooms (HCBs). https://hcb-1.itrcweb.org/.

[18] NEIWPCC (New England Interstate Water Pollution Control Commission) (2015). Harmful Algal Bloom Control Methods Synopses. http://www.neiwpcc.org/neiwpcc_docs/NEIWPCC_HABControlMethodsSynopses_June2015.pdf.

[19] Newcombe G., House J., Ho L., Baker P., Burch M. (2010). Management Strategies for Cyanobacteria (Blue-Green Algae): A Guide for Water Utilities.

[20] Rajasekhar P., Fan L., Nguyen T., Roddick F.A. (2012). A review of the use of sonication to control cyanobacterial blooms. Water Res..

[21] Lurling M., Waajen G., de Senerpoint Domis L.N. (2016). Evaluation of several end-of-pipe measures proposed to control cyanobacteria. Aquat. Ecol..

[22] Piel T., Sandrini G., White E., Xu T., Schuurmans J.M., Huisman J., Visser P.M. (2019). Suppressing Cyanobacteria with Hydrogen Peroxide Is More Effective at High Light Intensities. Toxins.

[23] Breda-Alves F., de Oliveira Fernandes V., Chia M.A. (2021). Understanding the environmental roles of herbicides on cyano-bacteria, cyanotoxins, and cyanoHABs. Aquat. Ecol..

[24] Sukenik A., Kaplan A. (2021). Cyanobacterial Harmful Algal Blooms in Aquatic Ecosystems: A Comprehensive Outlook on Current and Emerging Mitigation and Control Approaches. Microorganisms.

[25] Yoshida T., Takashima Y., Tomaru Y., Shirai Y., Takao Y., Hiroishi S., Nagasaki K. (2006). Isolation and Characterization of a Cyanophage Infecting the Toxic Cyanobacterium *Microcystis Aeruginosa*. Appl. Environ. Microbiol..

[26] Deng L., Hayes P.K. (2008). Evidence for cyanophages active against bloom-forming freshwater cyanobacteria. Freshw. Biol..

[27] Weinbauer M.G. (2004). Ecology of prokaryotic viruses. FEMS Microbiol. Rev..

[28] Singh P., Singh S.S., Srivastava A., Singh A., Mishra A.K. (2012). Structural, functional and molecular basis of cyanophage-cyanobacterial interactions and its significance. Afr. J. Biotechnol..

[29] Catalao M.J., Gil F., Moniz-Pereira J., São-José C., Pimentel M. (2013). Diversity in bacterial lysis systems: Bacteriophages show the way. FEMS Microbiol. Rev..

[30] Ortmann A., Lawrence J., Suttle C. (2002). Lysogeny and Lytic Viral Production during a Bloom of the Cyanobacterium *Synechococcus* spp.. Microb. Ecol..

[31] Jassim S.A.A., Limoges R.G. (2013). Impact of external forces on cyanophage–host interactions in aquatic ecosystems. World J. Microbiol. Biotechnol..

[32] Dorigo U., Jacquet S., Humbert J.-F. (2004). Cyanophage diversity, inferred from g20 gene analyses, in the largest natural lake in France, Lake Bourget. Appl. Environ. Microbiol..

[33] Gao E.-B., Huang Y., Ning D. (2016). Metabolic Genes within Cyanophage Genomes: Implications for Diversity and Evolution. Genes.

[34] Finke J.F., Suttle C.A. (2019). The Environment and Cyanophage Diversity: Insights from Environmental Sequencing of DNA Polymerase. Front. Microbiol..

[35] Safferman R., Cannon R., Desjardins P., Gromov B., Haselkorn R., Sherman L., Shilo M. (1983). Classification and Nomenclature of Viruses of Cyanobacteria. Intervirology.

[36] Safferman R.S., Morris M.-E. (1964). Growth characteristics of the blue-green algal virus LPP-1. J. Bacteriol..

[37] Padan E., Shilo M. (1973). Cyanophages-viruses attacking blue-green algae. Bacteriol. Rev..

[38] Xia H., Li T., Deng F., Hu Z. (2013). Freshwater cyanophages. Virol. Sin..

[39] Yoshida M., Yoshida T., Kashima A., Takashima Y., Hosoda N., Nagasaki K., Hiroishi S. (2008). Ecological Dynamics of the Toxic Bloom-Forming Cyanobacterium *Microcystis Aeruginosa* and Its Cyanophages in Freshwater. Appl. Environ. Microbiol..

[40] Morimoto D., Tominaga K., Nishimura Y., Yoshida N., Kimura S., Sako Y., Yoshida T. (2019). Coocurrence of broad- and narrow-host-range viruses infecting the bloom-forming toxic cyanobacterium *Microcystis Aeruginosa*. Appl. Environ. Microbiol..

[41] Sullivan M.B., Coleman M., Weigele P., Rohwer F., Chisholm S.W. (2005). Three Prochlorococcus Cyanophage Genomes: Signature Features and Ecological Interpretations. PLoS Biol..

[42] Yoshida T., Nagasaki K., Takashima Y., Shirai Y., Tomaru Y., Takao Y., Sakamoto S., Hiroishi S., Ogata H. (2008). Ma-LMM01 Infecting Toxic *Microcystis Aeruginosa* Illuminates Diverse Cyanophage Genome Strategies. J. Bacteriol..

[43] Wilson W.H., Joint I.R., Carr N.G., Mann N.H. (1993). Isolation and Molecular Characterization of Five Marine Cyanophages Propagated on *Synechococcus* sp. Strain WH7803. Appl. Environ. Microbiol..

[44] Wang K., Chen F. (2004). Genetic diversity and population dynamics of cyanophage communities in the Chesapeake Bay. Aquat. Microb. Ecol..

[45] Jakulska A., Mankiewicz-Boczek J. (2020). Cyanophages specific to cyanobacteria from the genus Microcystis. Int. J. Ecohydrol. Hydrobiol..

[46] Miskiewicz E., Ivanov A.G., Williams J.P., Khan M.U., Falk S., Huner N.P. (2000). Photosynthetic acclimation of the filamentous cyanobacterium, *Plectonema boryanum* UTEX 485, to temperature and light. Plant Cell Physiol..

[47] Paerl H.W. (2014). Mitigating Harmful Cyanobacterial Blooms in a Human- and Climatically-Impacted World. Life.

[48] Bratbak G., Heldal M., Norland S., Thingstad T.F. (1990). Viruses as Partners in Spring Bloom Microbial Trophodynamics. Appl. Environ. Microbiol..

[49] Suttle C.A., Chen F. (1992). Mechanisms and Rates of Decay of Marine Viruses in Seawater. Appl. Environ. Microbiol..

[50] Manage M.P., Kawabata Z., Nakano S.-I. (1999). Dynamics of cyanophage-like particles and algicidal bacteria causing *Microcystis Aeruginosa* mortality. Limnology.

[51] Safferman R., Schneider I., Steere R., Morris M., Diener T. (1969). Phycovirus SM-1: A virus infecting unicellular blue-green algae. Virology.

[52] Safferman R., Diener T., Desjardins P., Morris M. (1972). Isolation and characterization of AS-1, a phycovirus infecting the blue-green algae, Anacystis nidulans and Synechococcus cedrorum. Virology.

[53] Cheng K., Van de Waal D., Niu X.Y., Zhao Y.J. (2017). Combined Effects of Elevated pCO_2_ and Warming Facilitate Cyanophage Infections. Front. Microbiol..

[54] Murray A., Jackson G. (1992). Viral dynamics: A model of the effects of size shape, motion and abundance of single-celled olanktonic organisms and other particles. Mar. Ecol. Prog. Ser..

[55] Chu T.-C., Murray S.R., Hsu S., Vega Q., Lee L.H. (2011). Temperature-induced activation of freshwater cyanophage AS-1 prophage. Acta Histochem..

[56] Pick F.R., Lean D.R.S. (1987). The role of macronutrients (C, N, P) in controlling cyanobacterial dominance in temperate lakes. N. Z. J. Mar. Freshw. Res..

[57] Parrish J. (2014). The Role of Nitrogen and Phosphorus in the Growth, Toxicity, and Distribution of the Toxic Cyanobacteria *Microcystis Aeruginosa*. Master’s Projects and Capstones. https://repository.usfca.edu/capstone/8.

[58] Zachary A. (1978). An ecological study of bacteriophages of *Vibrio natriegens*. Can. J. Microbiol..

[59] Gobler C.J., Burkholder J.M., Davis T.W., Harke M.J., Johengen T., Stow C., Van de Waal D. (2016). The dual role of nitrogen supply in controlling the growth and toxicity of cyanobacterial blooms. Harmful Algae.

[60] Bulgakov N.G., Levich A.P. (1999). The nitrogen: Phosphorus ratio as a factor regulating phytoplankton community structure. Fundam. Appl. Limnol..

[61] Davidson K., Gowen R.J., Tett P., Bresnan E., Harrison P.J., McKinney A., Milligan S., Mills D.K., Silke J., Crooks A.M. (2012). Harmful algal blooms: How strong is the evidence that nutrient ratios and forms influence their occurrence?. Estuar. Coast. Shelf Sci..

[62] Davis W.T., Bullerjahn G.S., Tuttle T., McKay R.M., Watson S.B. (2015). Effects of increasing nitrogen and phosphorous concentrations on phytoplankton community growth and toxicity during Planktothrix blooms in Sandusky Bay, Lake Erie. Environ. Sci. Technol..

[63] Zimmerman A.E., Howard-Varona C., Needham D.M., John S.G., Worden A.Z., Sullivan M.B., Waldbauer J.R., Coleman M.L. (2019). Metabolic and biogeochemical consequences of viral infection in aquatic ecosystems. Nat. Rev. Microbiol..

[64] Waldbauer J.R., Coleman M.L., Rizzo A.I., Campbell K.L., Lotus J., Zhang L. (2019). Nitrogen sourcing during viral infection of marine cyanobacteria. Proc. Natl. Acad. Sci. USA.

[65] Wilson W.H., Carr N.G., Mann N.H. (1996). The effect of phosphate status on the kinetics of cyanophage infection in the oceanic *Cyanobacterium synechococcus* sp. WH78031. J. Phycol..

[66] Williamson S.J., Houchin L.A., McDaniel L., Paul J.H. (2002). Seasonal Variation in Lysogeny as Depicted by Prophage Induction in Tampa Bay, Florida. Appl. Environ. Microbiol..

[67] Rihtman B. (2016). Viral Infection of Marine Picoplankton under Nutrient Depletion Conditions: Pseudolysogeny and Magic Spot Nucleotides. Ph.D. Thesis.

[68] Zeng Q., Chisholm S. (2012). Marine viruses exploit their host’s two-component regulatory system in response to resource limitation. Curr. Biol..

[69] Mankiewicz-Boczek J., Jaskulska A., Pawełczyk J., Gągała I., Serwecińska L., Dziadek J. (2016). Cyanophages infection of Microcystis bloom in lowland dam reservoir of Sulejow, Poland. Microb. Ecol..

[70] Cheng K., Frenken T., Brussaard C.P.D., Van de Waal D. (2019). Cyanophage Propagation in the Freshwater *Cyanobacterium Phormidium* Is Constrained by Phosphorus Limitation and Enhanced by Elevated pCO_2_. Front. Microbiol..

[71] McKindles K. (2017). The Effect of Phosphorus and Nitrogen Limitation on Viral Infection in Microcystis Aeruginosa NIES298 Using the Cyanophage Ma-LMM01.

[72] McDaniel L., Paul J.H. (2004). Effect of nutrient addition and environmental factors on prophage induction in natural populations of marine *Synechococcus* species. Appl. Environ. Microbiol..

[73] Zhou Q., Gao Y., Zhao Y., Cheng K. (2015). The effect of elevated carbon dioxide concentration on cyanophage PP multiplication and photoreactivation induced by a wild host cyanobacterium. Acta Ecol. Sin..

[74] Verschoor M.J., Powe C.R., McQuay E., Schiff S.L., Venkiteswaran J.J., Li J., Molot L.A. (2017). Internal iron loading and warm temperatures are preconditions for cyanobacterial dominance in embayments along Georgian Bay, Great Lakes. Can. J. Fish. Aquat. Sci..

[75] Benson R., Martin E. (1984). Physicochemical characterization of cyanophage SM-2. Arch. Microbiol..

[76] Traving S.J., Clokie M.R., Middelboe M. (2013). Increased acidification has a profound effect on the interactions between the cyanobacterium *Synechococcus* sp. WH7803 and its viruses. FEMS Microbiol. Ecol..

[77] Davis T.W., Berry D.L., Boyer G.L., Gobler C.J. (2009). The effects of temperature and nutrients on the growth and dynamics of toxic and non-toxic strains of Microcystis during cyanobacteria blooms. Harmful Algae.

[78] Bozarth C.S., Schwartz A.D., Shepardson J.W., Colwell F.S., Dreher T.W. (2010). Population Turnover in a *Microcystis* Bloom Results in Predominantly Nontoxigenic Variants Late in the Season. Appl. Environ. Microbiol..

[79] Kardinaal W., Janse I., Agterveld M.K.-V., Meima M., Snoek J., Mur L., Huisman J., Zwart G., Visser P. (2007). Microcystis genotype succession in relation to microcystin concentrations in freshwater lakes. Aquat. Microb. Ecol..

[80] Zilliges Y., Kehr J.-C., Meissner S., Ishida K., Mikkat S., Hagemann M., Kaplan A., Börner T., Dittmann E. (2011). The Cyanobacterial Hepatotoxin Microcystin Binds to Proteins and Increases the Fitness of Microcystis under Oxidative Stress Conditions. PLoS ONE.

[81] Suttle C.A., Hurst C.J. (2000). 6—Ecological, evolutionary, and geochemical consequences of viral infection of cyanobacteria and eukaryotic algae. Viral Ecology.

[82] Cleaver J.E. (1974). IV—Photoreactivation. Adv. Radiat. Biol..

[83] Ni T., Zeng Q. (2016). Diel Infection of Cyanobacteria by Cyanophages. Front. Mar. Sci..

[84] Sherman L.A. (1976). Infection of Synechococcus cedrorum by the cyanophage AS-1M. III. Cellular metabolism and phage development. Virology.

[85] Mackenzie J.J., Haselkorn R. (1972). An electron microscope study of infection by the blue-green algal virus SM-1. Virology.

[86] Teklemariam A.T., Demeter S., Deak Z., Suryani G., Borebely G. (1990). AS-1 cyanophage infection inhibits the photosynthetic electron flow of photosystem II in *Synechococcus* sp. PCC 6301, a cyanobacterium. FEBS Lett..

[87] Yoshida-Takashima Y., Yoshida M., Ogata H., Nagasaki K., Hiroishi S., Yoshida T. (2012). Cyanophage Infection in the Bloom-Forming Cyanobacteria *Microcystis Aeruginosa* in Surface Freshwater. Microbes Environ..

[88] Nakamura G., Kimura S., Sako Y., Yoshida T. (2014). Genetic diversity of Microcystis cyanophages in two different freshwater environments. Arch. Microbiol..

[89] De Philippis R., Sili C., Paperi R., Vincenzini M. (2001). Exopolysaccharide-producing cyanobacteria and their possible exploitation: A review. J. Appl. Phycol..

[90] De Philippis R., Colica G., Micheletti E. (2011). Exopolysaccharide-producing cyanobacteria in heavy metal removal from water: Molecular basis and practical applicability of the biosorption process. Appl. Microbiol. Biotechnol..

[91] Kehr J.-C., Dittmann E. (2015). Biosynthesis and Function of Extracellular Glycans in Cyanobacteria. Life.

[92] Baulina O.I., Titel K., Gorelova O.A., Malai O.V., Ehwald R. (2008). Permeability of cyanobacterial mucous surface structures for macromolecules. Microbiology.

[93] Abedon S.T. (2017). Phage “delay” towards enhancing bacterial escape from biofilms: A more comprehensive way of viewing resistance to bacteriophages. AIMS Microbiol..

[94] Abedon S. (2016). Bacteriophage exploitation of bacterial biofilms: Phage preference for less mature targets?. FEMS Microbiol. Lett..

[95] Hughes K., Sutherland I., Clark J., Jones M. (1998). Bacteriophage and associated polysaccharide depolymerases—Novel tools for study of bacterial biofilms. J. Appl. Microbiol..

[96] Li S., Ou T., Zhang Q. (2013). Two virus-like particles that cause lytic infections in freshwater cyanobacteria. Virol. Sin..

[97] Jiang X., Ha C., Lee S., Kwon J., Cho H., Gorham T., Lee J. (2019). Characterization of Cyanophages in Lake Erie: Interaction Mechanisms and Structural Damage of Toxic Cyanobacteria. Toxins.

[98] Coello-Camba A., Diaz-Rua R., Duarte C.M., Irigoien X., Pearman J.K., Alam I.S., Agusti S. (2020). Picocyanobacteria Community and Cyanophage Infection Responses to Nutrient Enrichment in a Mesocosms Experiment in Oligotrophic Waters. Front. Microbiol..

[99] Safferman R.S., Morris M.E. (1963). Algal virus: Isolation. Science.

[100] World Health Organization (2003). Cyanobacterial Toxins: Microcystin-LR in Drinking Water.

[101] Phlips E.J., Zeman C., Hansen P. (1989). Growth, photosynthesis, nitrogen fixation and carbohydrate production by a unicellular cyanobacterium, *Synechococcus* sp. (Cyanophyta). J. Appl. Phycol..

[102] Huang W.-J., Cheng Y.-L., Cheng B.-L. (2008). Ozonation By-products and Determination of Extracellular Release in Freshwater Algae and Cyanobacteria. Environ. Eng. Sci..

[103] Beversdorf L.J., Miller T.R., McMahon K.D. (2013). The Role of Nitrogen Fixation in Cyanobacterial Bloom Toxicity in a Temperate, Eutrophic Lake. PLoS ONE.

[104] Rolff C., Almesjo L., Elmgren R. (2007). Nitrogen fixation and abundance of the diazotrophic cyanobacterium *Aphanizomenon* sp. in the Baltic Proper. MEPS.

[105] Karlson A.M.L., Duberg J., Motwani N.H., Hogfors H., Klawonn I., Ploug H., Svedén J.B., Garbaras A., Sundelin B., Hajdu S. (2015). Nitrogen fixation by cyanobacteria stimulates production in Baltic food webs. Ambio.

[106] Willis A., Chuang A.W., Woodhouse J.N., Neilan B.A., Burford M.A. (2016). Intraspecific variation in growth, morphology and toxin quotas for the cyanobacterium, *Cylindrospermopsis raciborskii*. Toxicon.

[107] Omoregie O.E., Crumbliss L.L., Bebout B.M., Zehr J.P. (2004). Determination of nitrogen-fixing phylotypes in *Lyngbya* sp. and Microcoleus chthonoplastes cyanobacterial mats from Guerrero Negro, Baja California, Mexico. Appl. Environ. Microbiol..

[108] Jones A.C., Monroe E.A., Podell S., Hess W.R., Klages S., Esquenazi E., Niessen S., Hoover H., Rothmann M., Lasken R.S. (2011). Genomic insights into the physiology and ecology of the marine filamentous cyanobacterium *Lyngbya majuscule*. Proc. Natl. Acad. Sci. USA.

[109] Pancrace C., Jokela J., Sassoon N., Ganneau C., Desnos-Ollivier M., Wahlsten M., Humisto A., Calteau A., Bay S., Fewer D.P. (2017). Rearranged biosynthetic gene cluster and synthesis of Hassalladin E in Planktothrix serta PCC 8927. ACS Chem. Biol..

[110] Fox J.A., Booth S., Martin E. (1976). Cyanophage SM-2: A new blue-green algal virus. Pathol. Microbiol..

[111] Tucker S., Pollard P. (2005). Identification of Cyanophage Ma-LBP and Infection of the Cyanobacterium *Microcystis Aeruginosa* from an Australian Subtropical Lake by the Virus. Appl. Environ. Microbiol..

[112] Hargreaves K.R., Anderson N.J., Clokie M.R. (2012). Recovery of viable cyanophages from the sediments of a eutrophic lake at decadal timescales. FEMS Microbiol. Ecol..

[113] Ou T., Li S., Liao X., Zhang Q. (2013). Cultivation and characterization of the MaMV-DC cyanophage that infects bloom-forming cyanobacterium *Microcystis Aeruginosa*. Virol. Sin..

[114] Wang J., Bai P., Li Q., Lin Y., Huo D., Ke F., Zhang Q., Li T., Zhao J. (2019). Interaction between cyanophage MaMV-DC and eight Microcystis strains, revealed by genetic defense systems. Harmful Algae.

[115] Currier T.C., Wolk C.P. (1979). Characteristics of Anabaena variabilis influencing plaque formation by cyanophage N-1. J. Bacteriol..

[116] Koz’yakov S.Y. (1977). Cyanophages of the series A(L) specific for the blue-green alga Anabaena variabilis. Exp. Algol. Biol. Sci. Res..

[117] Hu N.-T., Thiel T., Giddings T.H., Wolk C. (1981). New Anabaena and Nostoc cyanophages from sewage settling ponds. Virology.

[118] Monegue R.L., Phlips E.J. (1991). The effect of cyanophages on the growth and survival of *Lyngbya wollei*, *Anabaena flos-aquae*, and *Anabaena circinalis*. J. Aquat. Plant Manag..

[119] Granhall U. (1972). Aphanizomenon flos-aquae: Infection by Cyanophages. Physiol. Plant..

[120] Coulombe A.C., Robinson G.G.C. (1981). Collapsing *Aphanizomenon flos-aquae* blooms: Possible contributions of photo-oxidation, O_2_ toxicity, and cyanophages. Can. J. Bot..

[121] Šulčius S., Alzbutas G., Kvederavičiūtė K., Koreivienė J., Zakrys L., Lubys A., Paškauskas R. (2015). Draft genome sequence of the cyanobacterium Aphanizomenon flos-aquae strain 2012/KM1/DE isolated from the Curonian Lagoon (Baltic Sea). Genome Announc..

[122] Šulčius S., Slavuckytė K., Paškauskas R. (2017). The predation paradox: Synergistic and antagonistic interactions between grazing by crustacean predator and infection by cyanophages promotes bloom formation in filamentous cyanobacteria. Limnol. Oceanogr..

[123] Šulčius S., Šimoliūnas E., Alzbutas G., Gasiūnas G., Jauniškis V., Kuznecova J., Miettinen S., Nilsson E., Meškys R., Roine E. (2018). Genomic characterization of cyanophage vB_AphaS-CL131 infecting filamentous diazotrophic cyanobacterium Aphanizomenon flos-aquae reveals novel insights into virus-bacterium interactions. Appl. Environ. Microbiol..

[124] Singh R.N., Singh P.K.S.R.N. (1967). Isolation of Cyanophages from India. Nature.

[125] Pollard P., Young L.M. (2010). Lake viruses lyse cyanobacteria, Cylindrospermopsis raciborskii, enhances filamentous-host dispersal in Australia. Acta Oecologica Int. J. Ecol..

[126] Steenhauer M.L., Wierenga J., Carreira C., Limpens R.W.A.L., Koster A.J. (2016). Isolation of cyanophage CrV infecting Cylin-drospermopsis raciborskii and the influence of temperature and irradiance on CrV proliferation. Aquat. Microb. Ecol..

[127] Gao E., Yuan X., Li R., Zhang Q. (2009). Isolation of a novel cyanophage infectious to the filamentous cyanobacterium *Planktothrix agardhii* (Cyanophyceae) from Lake Donghu, China. Aquat. Microb. Ecol..

[128] Watkins C.S., Smith J.R., Hayes P.K., Watts J.E.M. (2014). Characterisation of host growth after infection with a broad-range freshwater cyanopodophage. PLoS ONE.

[129] Hewson I., O’Neil J.M., Dennison W.C. (2001). Virus-like particles associated with Lyngbya icocyano (Cyanophyta; Oscillatoriacea) bloom decline in Moreton Bay, Australia. Aquat. Microb. Ecol..

[130] Fahnensteil G.L., Carrick H.J. (1991). Physiological characteristics and food-web dynamics of Synechococcus in Lakes Huron and Michigan. Limnol. Oceanogr..

[131] Scanlan J.D., West N.J. (2002). Molecular ecology of the marine cyanobacterial genera Prochlorococcus and Synechococcus. FEMS Microbiol. Ecol..

[132] Callieri C., Cronberg G., Stockner J., Whitton B. (2012). Freshwater icocyanobacterial: Single cells, microcolonies and colonial forms. Ecology of Cyanobacteria II: Their Diversity in Time and Space.

[133] Mitsui A., Cao S., Takahashi A., Arai T. (1987). Growth synchrony and cellular parameters of the unicellular nitrogen-fixing marine cyanobacterium, *Synechococcus* sp. strain Miami BG 043511 under continuous illumination. Physiol. Plant..

[134] Steunou A.-S., Bhaya D., Bateson M.M., Melendrez M.C., Ward D.M., Brecht E., Peters J.W., Kühl M., Grossman A.R. (2006). *In situ* analysis of nitrogen fixation and metabolic switching in unicellular thermophilic cyanobacteria inhabiting hot spring microbial mats. Proc. Natl. Acad. Sci. USA.

[135] Blaha L., Marsalek B. (1999). Microcystin production and toxicity of picocyanobacterial as risk factor for drinking water treatment plants. Algol. Stud..

[136] Carmichael W.W., Li R. (2006). Cyanobacteria toxins in the Salton Sea. Aquat. Biosyst..

[137] Furtado A.L.F.F., Calijuri M.D.C., Lorenzi A.S., Honda R.Y., Genuário D.B., Fiore M.F. (2009). Morphological and molecular characterization of cyanobacteria from a Brazilian facultative wastewater stabilization pond and evaluation of microcystin production. Hydrobiologia.

[138] Kao C.C., Green S., Stein B., Golden S.S. (2005). Diel Infection of a Cyanobacterium by a Contractile Bacteriophage. Appl. Environ. Microbiol..

[139] Sherman L.A., Connelly M., Sherman D.M. (1976). Infection of Synechococcus cedrorum by the cyanophage AS-1M. I. Ultrastructure of infection and phage assembly. Virology.

[140] Sherman L.A., Pauw P. (1976). Infection of Synechococcus cedrorum by the cyanophage AS-1M. II. Protein and DNA synthesis. Virology.

[141] Kim M., Choi Y.-K. (1994). A New Synechococcus Cyanophage from a Reservoir in Korea. Virology.

[142] Park G.J., Kim M., Choi Y.K., Yoon S.N. (1996). Restriction pattern of the nucleic acid of *Synechococcus* sp. cyanophage. J. Microbiol..

[143] Dreher T.W., Brown N., Bozarth C.S., Schwartz A.D., Riscoe E., Thrash C., Bennett S.E., Tzeng S.-C., Maier C.S. (2011). A freshwater cyanophage whose genome indicates close relationships to photosynthetic marine cyanomyophages. Environ. Microbiol..

[144] Chénard C., Chan A.M., Vincent W., Suttle A.C. (2015). Polar freshwater cyanophage S-EIV1 represents a new widespread evolutionary lineage of phages. ISME J..

[145] Zhong K.X., Suttle C.A., Baudoux A.-C., Derelle E., Colombet J., Cho A., Caleta J., Six C., Jacquet S. (2018). A New Freshwater Cyanosiphovirus Harboring Integrase. Front. Microbiol..

[146] Yamamoto Y., Shiah F.-K., Chen Y.-L. (2011). Importance of large colony formation in bloom-forming cyanobacteria to dominate in eutrophic ponds. Ann. Limnol. Int. J. Limnol..

[147] Eldridge S.L.C., Wood T.M., Echols K.R. (2012). Spatial and Temporal Dynamics of Cyanotoxins and Their Relation to Other Water Quality Variables in Upper Klamath Lake, Oregon, 2007–2009.

[148] Welker M., von Dohren H. (2006). Cyanobacterial peptides—Nature’s own combinatorial synthesis. FEMS Microbiol. Rev..

[149] Otten G.T., Paerl H.W. (2015). Health effects of toxic cyanobacteria in US drinking and recreational waters: Our current understanding and proposed direction. Water Health.

[150] Takashima Y., Yoshida T., Yoshida M., Shirai Y., Tomaru Y., Takao Y., Hiroishi S., Nagasaki K. (2007). Development and Application of Quantitative Detection of Cyanophages Phylogenetically Related to Cyanophage Ma-LMM01 Infecting *Microcystis Aeruginosa* in Fresh Water. Microbes Environ..

[151] Kimura-Sakai S., Sako Y., Yoshida T. (2015). Development of a real-time PCR assay for the quantification of Ma-LMM01-type Microcystis cyanophages in a natural pond. Lett. Appl. Microbiol..

[152] Kimura S., Yoshida T., Hosoda N., Honda T., Kuno S., Kamiji R., Hashimoto R., Sako Y. (2012). Diurnal infection patterns and impact of Microcystis cyanophages in a Japanese pond. Appl. Environ. Microbiol..

[153] Morimoto D., Kimura S., Sako Y., Yoshida T. (2018). Transcriptome Analysis of a Bloom-Forming Cyanobacterium *Microcystis Aeruginosa* during Ma-LMM01 Phage Infection. Front. Microbiol..

[154] Ou T., Gao X.-C., Li S.-H., Zhang Q.-Y. (2015). Genome analysis and gene nblA identification of *Microcystis Aeruginosa* myovirus (MaMV-DC) reveal the evidence for horizontal gene transfer events between cyanomyovirus and host. J. Gen. Virol..

[155] Komarek J., Kovacik L. (1989). Trichome structure of four *Aphanizomenon taxa* (Cyanophyceae) from Czechoslovakia, with notes on the taxonomy and delimitation of the genus. Plant Syst. Evol..

[156] Gugger M., Lyra C., Henriksen P., Couté A., Humbert J.-F., Sivonen K. (2002). Phylogenetic comparison of the cyanobacterial genera Anabaena and Aphanizomenon. Int. J. Syst. Evol. Microbiol..

[157] Kipp R.M. (2006). Cylindrospermopsis Raciborskii Factsheet. https://www.glerl.noaa.gov/res/HABs_and_Hypoxia/cylindro_factsheet.html.

[158] Sivonen K., Börner T., Herrero A., Flores E. (2008). Bioactive compounds produced by cyanobacteria. The Cyanobacteria: Molecular Biology, Genomics and Evolution.

[159] Bancroft I., Smith R.J. (1988). The isolation of genomic DNA from cyanophage infecting Nostoc and Anabaena species of cyanobacteria. New Phytol..

[160] Bancroft I., Smith R.J. (1988). An analysis of restriction endonuclease sites in cyanophages infecting the heterocystous cyanobacteria Anabaena and Nostoc. J. Gen. Virol..

[161] Bancroft I., Wolk C.P., Oren E.V. (1989). Physical and genetic maps of the genome of the heterocyst-forming cyanobacterium *Anabaena* sp. strain PCC 7120. J. Bacteriol..

[162] Baker A.C., Goddard V.J., Davy J., Schroeder D.C., Adams D.G., Wilson W.H. (2006). Identification of a Diagnostic Marker To Detect Freshwater Cyanophages of Filamentous Cyanobacteria. Appl. Environ. Microbiol..

[163] Xiong Z., Wang Y., Dong Y., Zhang Q., Xu X. (2019). Cyanophage A-1(L) adsorbs to lipopolysaccharides of Anabaena sp. strain PCC7120 via the tail protein lipopolysaccharide-interacting protein (ORF36). J. Bacteriol..

[164] Wu W., Zhu Q., Liu X., An C., Wang J. (2009). Isolation of a freshwater cyanophage (F1) capable of infecting Anabaena flos-aquae and its potentials in the control of water bloom. Int. J. Environ. Pollut..

[165] Salam E.A., Shabana E.T., Din A.M. (2014). Isolation and characterization of two cyanophages infecting some Anabaena spp.. Egypt. J. Biol. Pest Control..

[166] Kurmayer R., Deng L., Entfellner E. (2016). Role of toxic and bioactive secondary metabolites in colonization and bloom formation by filamentous cyanobacteria Planktothrix. Harmful Algae.

[167] Sharp K., Arthur K.E., Gu L., Ross C., Harrison G., Gunasekera S.P., Meickle T., Matthew S., Luesch H., Thacker R.W. (2009). Phylogenetic and Chemical Diversity of Three Chemotypes of Bloom-Forming *Lyngbya* Species (*Cyanobacteria*: *Oscillatoriales*) from Reefs of Southeastern Florida. Appl. Environ. Microbiol..

[168] Paul J.V., Cruz-Rivera E., Thacker R.W., McClintock J., Baker B. (2001). Chemical mediation of macroalgal-herbivore interactions: Ecological and evolutionary perspectives. Marine Chemical Ecology.

[169] Suda S., Watanabe M.M., Otsuka S., Mahakahant A., Yongmanitchai W., Nopartnaraporn N., Liu Y., Day J.G. (2002). Taxonomic revision of water-bloom-forming species of oscillatorioid cyanobacteria. Int. J. Syst. Evol. Microbiol..

[170] Bratbak G., Jacobsen A., Heldal M., Nagasaki K., Thingstad F. (1998). Virus production in Phaeocystis pouchetii and its relation to host cell growth and nutrition. Aquat. Microb. Ecol..

[171] Zborowsky S., Lindell D. (2019). Resistance in marine cyanobacteria differs against specialist and generalist cyanophages. Proc. Natl. Acad. Sci. USA.

[172] Jia Y., Shan J., Millard A., Clokie M.R., Mann N.H. (2010). Light-dependent adsorption of photosynthetic cyanophages to Synechococcus sp. WH7803. FEMS Microbiol. Lett..

[173] Mangan N.M., Flamholz A., Hood R.D., Milo R., Savage D.F. (2016). pH determines the energetic efficiency of the cyanobacterial CO _2_ concentrating mechanism. Proc. Natl. Acad. Sci. USA.

[174] Stanier R.Y., Kunisawa R., Mandel M., Cohen-Bazire G. (1971). Purification and properties of unicellular blue-green algae (order Chroococcales). Bacteriol. Rev..

[175] Waechter C., Aligata A., Zhang Y. (2019). Viral Treatment of Harmful Algal Blooms.

